# Rapid identification of capsulated *Acinetobacter baumannii* using a density-dependent gradient test

**DOI:** 10.1186/s12866-020-01971-9

**Published:** 2020-09-16

**Authors:** Hadas Kon, David Schwartz, Elizabeth Temkin, Yehuda Carmeli, Jonathan Lellouche

**Affiliations:** 1 National Institute for Antibiotic Resistance and Infection Control, Ministry of Health, Tel-Aviv Sourasky Medical Center, 6 Weizmann St, 6423906 Tel-Aviv, Israel; 2 Sackler Faculty of Medicine, Tel-Aviv University, Tel-Aviv, Israel

**Keywords:** Carbapenem-resistant *Acinetobacter baumannii*, Capsule, Mucoid phenotype, Hypervirulence

## Abstract

**Background:**

Gram-negative bacterial capsules are associated with production of carbohydrates, frequently resulting in a mucoid phenotype. Infections caused by capsulated or mucoid *A. baumannii* are associated with increased clinical severity. Therefore, it is clinically and epidemiologically important to identify capsulated *A. baumannii.* Here, we describe a density-dependent gradient test to distinguish between capsulated and thin/non-capsulated *A. baumannii*.

**Results:**

Thirty-one of 57 *A. baumannii* isolates displayed a mucoid phenotype. The density-dependent gradient test was comprised of two phases, with silica concentrations of 30% (top phase) and 50% (bottom phase). Twenty-three isolates migrated to the bottom phase, indicating thin or non-capsulated strains, and 34 migrated to the top phase, suggesting strains suspected to be capsulated. There was agreement between the mucoid and the non-mucoid phenotypes and the density-dependent gradient test for all but three isolates. Total carbohydrates extracted from strains suspected to be capsulated were significantly higher. Transmission electron microscopy confirmed the presence of a capsule in the six representative strains suspected to be capsulated.

**Conclusions:**

The density-dependent gradient test can be used to verify capsule presence in mucoid-appearing *A. baumannii* strains. Identifying capsulated strains can be useful for directing infection control measures to reduce the spread of hypervirulent strains.

## Background

*Acinetobacter baumannii* is a major cause of hospital-acquired infections [1]. *A. baumannii* virulence factors include siderophore-mediated iron acquisition systems, biofilm formation, motility, and a remarkable capacity to acquire and rearrange genetic determinants [2]. These virulence factors are involved in the pathobiology and infection process, such as binding to host epithelial cells, cellular damage, serum resistance, and invasion [3]. Surface carbohydrates are known to influence virulence and fitness of *A. baumannii*. These carbohydrates include capsular polysaccharides (capsule), lipooligosaccharide (LOS) and the exopolysaccharide poly-β-(1–6)-N-acetylglucosamine (PNAG). While surface carbohydrates influence pathogenicity, studies show that the capsule is the predominant virulence factor in *A. baumann*ii [4, 5]. Capsules are high-molecular-weight hydrophilic polymers that form a layer enveloping the bacterial cell. In *A. baumannii*, capsules are composed of tightly packed repeating oligosaccharide subunits (K units), typically consisting of 4–6 sugars [5, 6]. Capsules provide a protective shield against immune recognition by limiting interactions between immunogenic surface structures of the pathogen and host defenses, leading to immune evasion and serum resistance [5]. In addition, capsule production contributes to anti-phagocytic and anti-bacteriolytic activity, as the negatively charged and unique surface of the capsule prevents phagocytes from adhering [7].

Few methods are available to quantify surface carbohydrates and to determine their composition. Carbohydrate quantification can be obtained by uronic acid assay [8] and composition can be assessed by nuclear magnetic resonance or liquid chromatography [6, 9]. Clinical laboratories do not incorporate these methods because they are cumbersome and require hazardous materials.

Overproduction of surface carbohydrates comprising the bacterial capsule may result in a mucoid phenotype [10–12]. This phenotype may vary, depending on the growth medium, incubation time, and conditions [10–12]. The observed phenotype is subjective and difficult to determine by simple examination, thus requiring further confirmation. The string test is based on measuring the length of a thread-like mucoid that is formed from a loop drawn gradually away from the tested suspension. This test is commonly used by clinical laboratories for *Klebsiella pneumoniae* and *Vibrio cholera* isolates [13, 14]. Additionally, microscopy using India ink staining is performed to detect capsulated strains in *K. pneumoniae* and *Streptococcus* spp. [15]. No specific test exists to detect capsulated *A. baumannii*.

The density-dependent gradient method is a technique used to separate cells by size [16]. It is based on the migration of cells by centrifugation through a gradient matrix. The matrix is made up of multiple phases of an organic polymer, each phase consisting of a different polymer concentration, resulting in a gradient with various densities. This method is commonly used to purify different eukaryotic cell types [17], but is seldom used for bacteria. A few studies have used this method to separate strains of a specific bacteria based on capsule size [18–21]. Here, we describe a density-dependent gradient test that we developed to distinguish between capsulated and thin/non-capsulated *A. baumannii* using colloidal silica particles as a matrix.

## Results

### Phenotype categorization

**Fig. 1:**
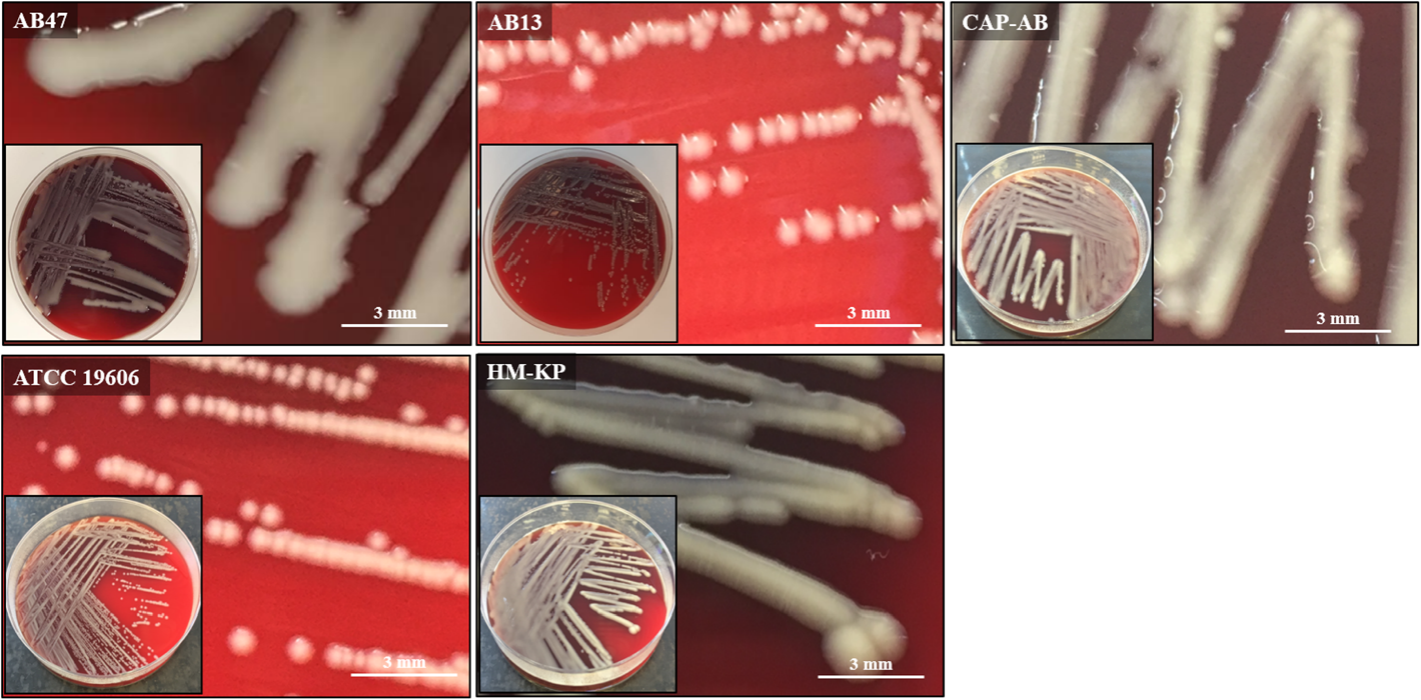
Phenotype of mucoid and non-mucoid isolates. Representative *A. baumannii* isolates with a mucoid (AB47) and a non-mucoid phenotype (AB13) observed on blood agar, following 18 h aerobic incubation at 35 ± 2 °C. The CAP-AB (capsulated and mucoid *A. baumannii*), ATCC 19606 and HM-KP (hypermucoid string test-positive *K. pneumoniae*) were used as controls

Of the 57 *A. baumannii* isolates, 31 (54.4%) displayed a mucoid phenotype and 26 (45.6%) a non-mucoid phenotype (Table 1). Colonies of mucoid *A. baumannii* appeared moist, raised, and viscid with irregular margins, while non-mucoid strains displayed a typical phenotype of small, round, convex colonies with distinct margins. The control strain ATCC 19606 displayed a non-mucoid phenotype as expected. The two other control strains, a hypermucoid string test-positive *K. pneumoniae* (HM-KP) and a mucoid *A. baumannii* with a significant capsule and an extracellular slime (CAP-AB), displayed a mucoid phenotype as expected (Fig. 1).

**Table 1:** Characteristics of mucoid and non-mucoid *A. baumannii* isolates and summary of results

	Sample	Origin	Clonal complex^**1**^	Density-dependent gradient^**2**^	Total carbohydrates (μg/mL)	Capsule thickness (nm)^**3**^
**Non-mucoid**^**4**^	AB01	Sputum	ST3	Non-capsulated^7^	44.0 ± 1.67	–
	AB02	Blood	ST3	Non-capsulated	35.3 ± 0.79	–
	AB03*	Blood	ST2	Non-capsulated	53.7 ± 2.45	–
	AB04	Rectal	ST3	Non-capsulated	74.8 ± 2.27	–
	AB05	Skin	ST3	Non-capsulated	83.7 ± 2.11	–
	AB06	Blood	N/D^5^	Non-capsulated	66.5 ± 1.39	–
	AB07	Blood	N/D	Non-capsulated	84.5 ± 2.24	–
	AB08	Blood	ST2	Non-capsulated	75.9 ± 1.24	–
	AB09	Sputum	ST3	Non-capsulated	39.0 ± 10.81	–
	AB10*	Blood	N/D	Non-capsulated	43.1 ± 2.20	–
	AB11	Sputum	ST3	Non-capsulated	54.8 ± 2.02	0
	AB12	Blood	ST2	Non-capsulated	55.2 ± 1.56	–
	AB13	Blood	N/D	Non-capsulated	64.5 ± 2.01	–
	AB14	Rectal	ST2	Non-capsulated	63.6 ± 1.78	–
	AB15	Skin	ST2	Non-capsulated	84.4 ± 2.54	–
	AB16	Blood	ST2	Non-capsulated	72.3 ± 1.56	–
	AB17	Blood	ST3	Non-capsulated	64.4 ± 2.27	–
	AB18	Blood	N/D	Non-capsulated	45.0 ± 1.56	–
	AB19	Pus	ST2	Non-capsulated	56.6 ± 1.65	–
	AB20	Blood	ST3	Non-capsulated	65.4 ± 2.43	–
	AB21	Blood	N/D	Non-capsulated	28.0 ± 4.15	–
	AB22	Skin	ST2	Non-capsulated	49.1 ± 1.79	–
	AB23	Wound	ST2	Non-capsulated	51.5 ± 1.59	–
	AB24	Sputum	ST3	Capsulated	254.3 ± 5.65	–
	AB25	Sputum	ST3	Capsulated	262.2 ± 12.2	–
	AB26	Blood	ST3	Capsulated	257.8 ± 5.48	–
	ATCC 19606*	Urine	ST52	Non-capsulated	34.5 ± 1.66	0
**Mucoid**^**4**^	AB27	Skin	ST3	Capsulated	212.8 ± 8.77	–
	AB28	Skin	ST3	Capsulated	426.5 ± 6.39	–
	AB29*	Blood	ST3	Capsulated	356.4 ± 4.61	–
	AB30	Wound	ST3	Capsulated	351.2 ± 5.44	–
	AB31	Blood	ST3	Capsulated	191.4 ± 6.14	–
	AB32	Sputum	ST3	Capsulated	240.0 ± 14.97	–
	AB33	Skin	ST3	Capsulated	412.0 ± 9.25	–
	AB34*	Skin	ST3	Capsulated	356.8 ± 7.85	92 ± 8
	AB35	Blood	ST3	Capsulated	317.3 ± 8.51	–
	AB36	Rectal	ST3	Capsulated	286.7 ± 8.98	–
	AB37	Rectal	ST3	Capsulated	290.6 ± 9.44	–
	AB38	Blood	ST3	Capsulated	166.7 ± 7.59	–
	AB39	Blood	ST3	Capsulated	275.2 ± 6.66	–
	AB40	Sputum	ST2	Capsulated	319.4 ± 9.78	–
	AB41	Blood	ST3	Capsulated	132.9 ± 7.32	–
	AB42	Skin	ST3	Capsulated	338.4 ± 11.11	–
	AB43	Blood	ST3	Capsulated	247.4 ± 7.40	122 ± 6
	AB44	Blood	ST3	Capsulated	261.0 ± 5.58	92 ± 13
	AB45*	Blood	N/D	Capsulated	207.7 ± 5.39	–
	AB46	Blood	ST3	Capsulated	364.7 ± 16.48	–
	AB47	Wound	ST3	Capsulated	316.6 ± 8.47	–
	AB48*	Blood	ST3	Capsulated	174.9 ± 40.31	–
	AB49	Wound	N/D	Capsulated	157.6 ± 6.37	–
	AB50	Tracheal aspirate	N/D	Capsulated	226.5 ± 17.51	–
	AB51	Tracheal aspirate	N/D	Capsulated	254.1 ± 9.04	–
	AB52	Tracheal aspirate	N/D	Capsulated	182.2 ± 9.45	–
	AB53	Sputum	ST3	Capsulated	264.2 ± 13.25	–
	AB54	Sputum	ST3	Capsulated	353.4 ± 15.07	–
	AB55	BAL^6^	N/D	Capsulated	132.3 ± 12.64	–
	AB56	Sputum	N/D	Capsulated	118.5 ± 16.17	–
	AB57	Sputum	N/D	Capsulated	130.7 ± 10.01	–
	CAP-AB*	Blood	ST3	Capsulated	290.6 ± 9.53	96 ± 11
	HM-KP*	Liver Abscess	K1	Capsulated	–	–

^1^Pasteur scheme; ^2^Length of migration: thin or non-capsulated if > 16 mm, suspected as capsulated if 0–16 mm; ^3^Determined by TEM; ^4^Determined by visual observation; ^5^Not determined; ^6^Bronchoalveolar lavage; ^7^Non-capsulated refers to an isolate with a thin capsule or no capsule; *Isolates used for validation of the density-dependent gradient test

### Determination of optimal parameters of the gradient composition

**Fig. 2:**
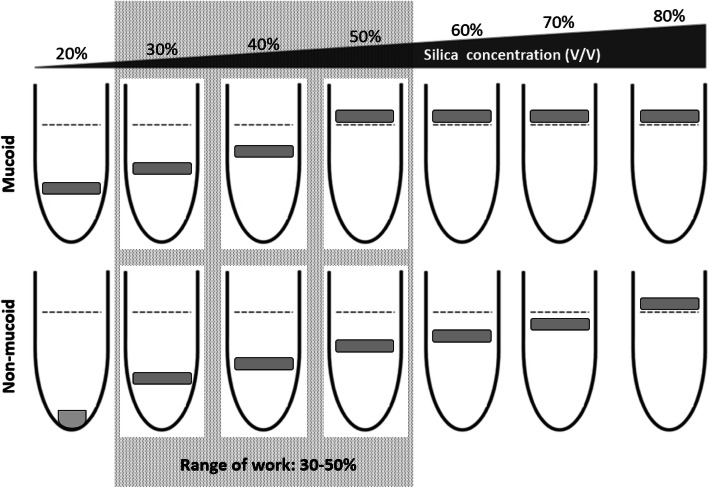
Validation of gradient composition. Schematic illustration of the method used to determine the optimal parameters of the gradient composition and the adequate range of work (box with dashed gray background). The range of work was determined by testing a panel of seven single silica concentrations (20–80% V/V). The validation was performed on three control strains (CAP-AB, HM-KP, ATCC 19606) and confirmed on six isolates selected randomly from the sample. The grey square illustrates a band of bacterial cells following centrifugation. The dashed line represents the upper liquid level of the silica sample

The bacterial bands of HM-KP and CAP-AB migrated successfully into the silica concentrations of 20, 30 and 40% (V/V) but were unable to penetrate the density of 50% or higher. Therefore, we selected 50% as the maximum phase concentration of the gradient matrix. The band of the non-mucoid control (ATCC 19606) migrated in a concentration range of 20–70%. The 20% concentration was rejected because the band settled at the bottom of the tube. Therefore, 30% was the minimum silica concentration of the phase range that a non-mucoid control strain could penetrate and be easily visualized. Thus, 30% was selected as the minimal phase concentration. The four mucoid and the two non-mucoid representative isolates displayed similar results to HM-KP/CAP-AB and ATCC 19606, respectively (Table 1). Thus, for our sample and validation settings, the range of work was 30–50% (Fig. 2).

**Fig. 3:**
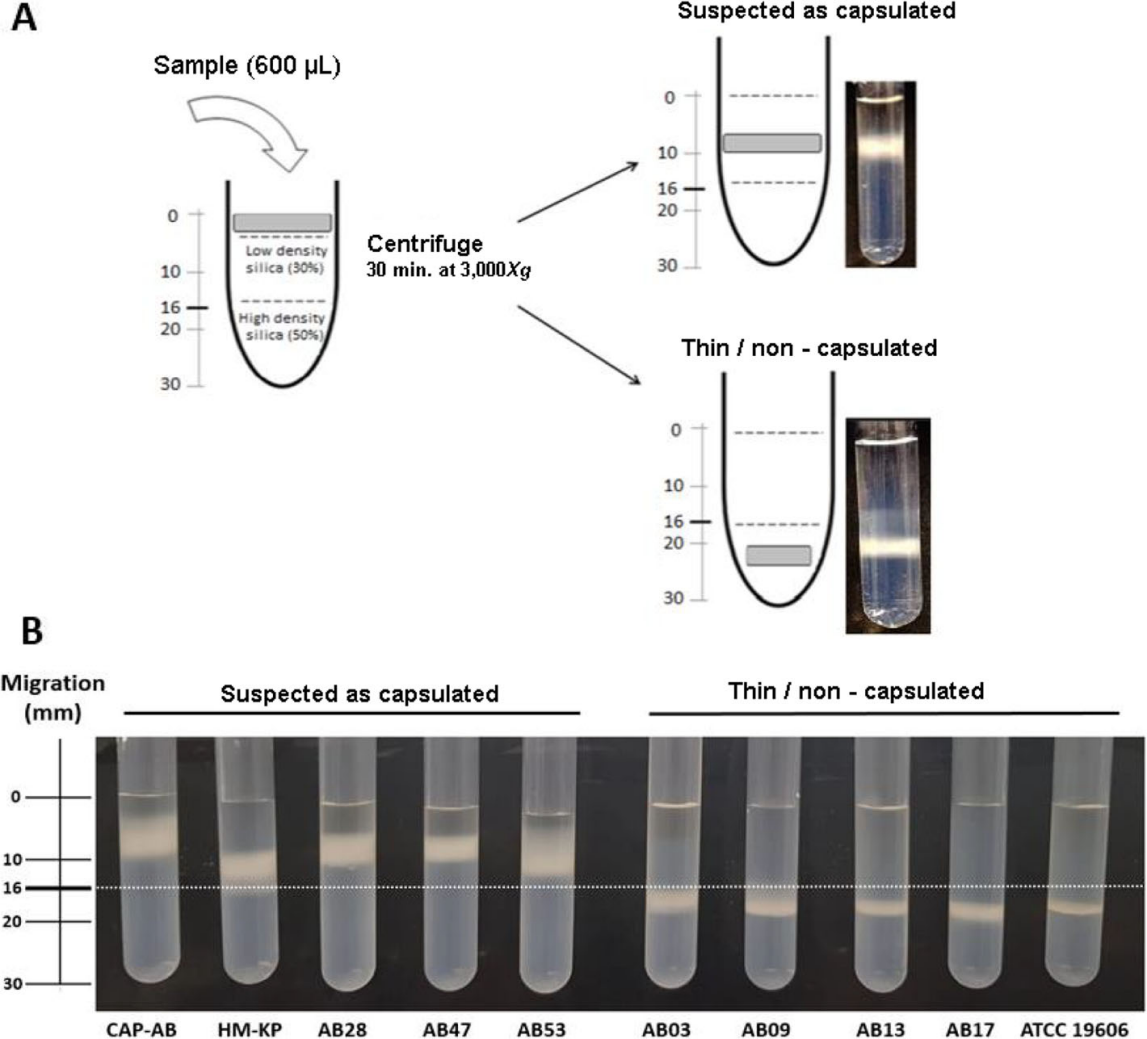
**a** Schematic illustration of the density-dependent gradient test. Bacterial cells (gray square) are applied to the top of a gradient matrix fixed in two phases (30 and 50% silica concentration). Following centrifugation, the bacterial cells migrate to either the top phase of the gradient (0 to 16 mm), indicating that the strain may be capsulated, or to the bottom phase (> 16 mm), indicating a thin or non-capsulated strain. **b** Density-dependent gradient test results. On the left are the two capsulated control strains (CAP-AB and HM-KP) and three representative isolates suspected to be capsulated, with bacterial bands in the top phase (0–16 mm). On the right is the control strain ATCC 19606 and four representative thin/non capsulated isolates with bands in the bottom phase (> 16 mm)

The final configuration of our test was a matrix with a volume of 2 mL composed of a 30% top phase (1 mL) and a 50% bottom phase (1 mL). After the addition of the bacterial inoculum (600 μL) and centrifugation, an isolate was suspected to be capsulated if the band migrated within 0–16 mm (top phase). Isolates were considered thin or non-capsulated if the band migrated to > 16 mm (bottom phase) (Fig. 3a). In this configuration, it is not possible to distinguish between an isolate with a thin capsule and a non-capsulated isolate, since they both migrate to the bottom phase.

### Density-dependent gradient test

Twenty-three of the 57 isolates (40.4%) migrated to the bottom phase, indicating thin or non-capsulated strains. Thirty-four isolates (59.6%) migrated within the top phase and were suspected to be capsulated (Fig. 3b and Table 1). For 54 isolates (94.7%), there was complete agreement between the density-dependent gradient test results and the phenotype determination. Three isolates (5.3%) exhibiting a non-mucoid phenotype were classified as capsulated by the density-dependent gradient test (isolates AB24, AB25, AB26). The strains used as controls exhibited expected results: HM-KP and CAP-AB displayed a band in the top phase, while ATCC 19606 migrated to the bottom phase (Fig. 3b).

**Fig. 4:**
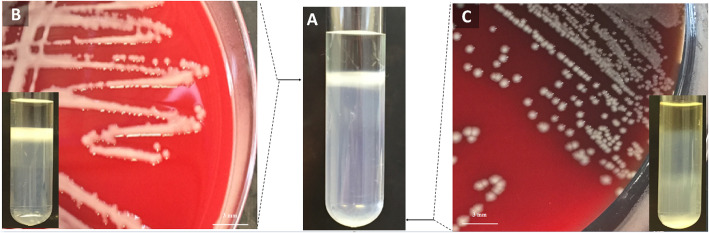
Heterogeneous culture. **a** Density-dependent gradient test of a heterogeneous culture with bacterial bands in both the top and bottom phases. **b** Isolate AB41 with a mucoid phenotype and its density-dependent gradient test (insert in B) with a single band in the top phase. **c** Isolate AB20 with a non-mucoid phenotype and its density-dependent gradient test (insert in C) with a single band settled in the bottom phase

One strain initially exhibited a band in both the top phase and the bottom phase, suggesting the presence of two different strains. Following further isolation, both strains were identified as *A. baumannii* (AB41 and AB20). Strain AB41 appeared to be mucoid and its band was observed on the top phase of the gradient, while strain AB20 displayed a non-mucoid phenotype and migrated to the bottom phase, signifying that it was thin/non-capsulated (Fig. 4). Molecular genotyping suggested that both isolates were identical and harbored a *bla*_OXA-374_ gene, a variant of the *bla*_OXA-71-LIKE_ gene. The distinct capsular phenotypes of different colonies of this strain may be due to phase variation.

When we repeated the density-dependent gradient test using isolates taken directly from agar plates, the phenotypes were identical to those obtained from the isolates prepared from brain heart infusion broth (BHI), including the three isolates that exhibited discrepancies (AB24, AB25, AB26).

### Carbohydrate production

**Fig. 5:**
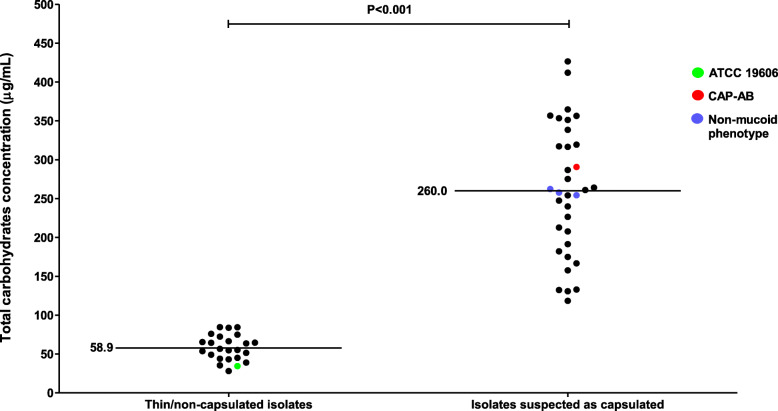
Total carbohydrates of thin or non-capsulated *A. baumannii* and of isolates suspected to be capsulated *A. baumannii*. Each black dot represents the carbohydrates average of six replicates for each isolate

In order to confirm capsule presence, we quantified the total polysaccharides present in the bacterial cell, including capsular polysaccharides and LOS. Among the 23 isolates classified as non-mucoid and thin/non-capsulated, the mean value of total carbohydrates was 58.9 ± 2.4 μg/mL. The carbohydrate amount measured for ATCC 19606 fell in this range (34.5 ± 1.7 μg/mL). In contrast, among the isolates that were mucoid and suspected to be capsulated, the mean carbohydrate amount was 260.0 ± 10.3 μg/mL. The value of total carbohydrates measured for CAP-AB fell in this range (290.6 ± 9.5 μg/mL). The difference between the thin/non-capsulated group and the group suspected to be capsulated was significant (*P* < 0.001). The carbohydrate amounts for the three isolates with discrepant phenotypes (mucoidicity and density-dependent gradient) were similar to the isolates suspected to be capsulated: 254.3 ± 5.7, 262.2 ± 12.2 and 257.8 ± 5.5 μg/mL (Fig. 5 and Table 1).

### Transmission electron microscopy (TEM) results

**Fig. 6:**
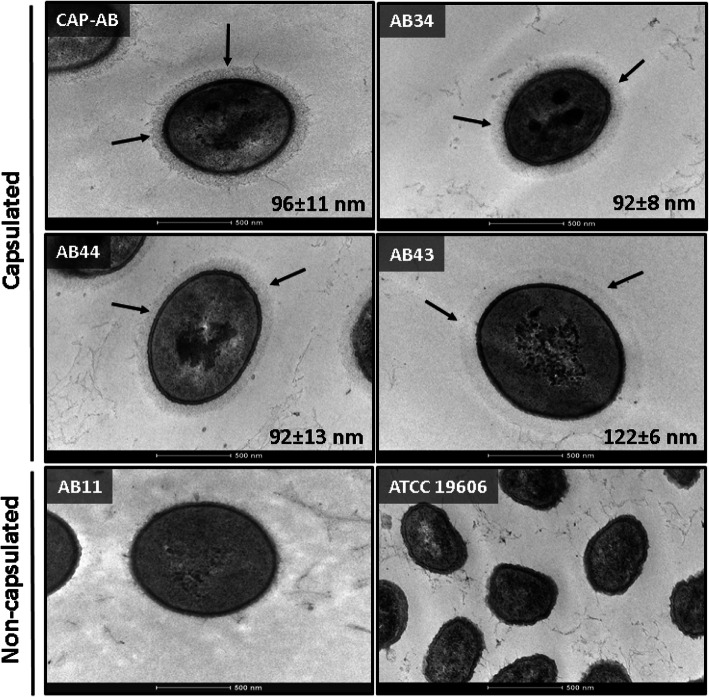
Capsule thickness. TEM images of capsulated control strain CAP-AB, representative capsulated isolates (AB34, AB44, AB43), a representative non-capsulated isolate (AB11), and thin-capsulated control strain ATCC 19606. The halo surrounding the cells of the capsulated isolates represents the capsule (black arrows). The numbers indicate the mean capsule thickness (nm) and standard deviation Magnification: 43000 X. scale bar: 500 nm

The presence of capsules was confirmed by TEM imaging. We analyzed three isolates that were mucoid and likely capsulated. All three displayed a significant polysaccharide matrix that was physically associated with the cell surface. The capsule was clearly visible as a light grey halo surrounding the outer membrane of the bacterial cell, associated with filaments of extracellular polysaccharides (Fig. 6). The mean capsule thickness of each isolate was 92 ± 8 (AB34), 92 ± 13 (AB44) and 122 ± 6 nm (AB43). The range of thickness values for all three isolates overlapped the range of capsule thickness of CAP-AB (96 ± 11 nm) (Fig. 6). No capsule was visible in the images of an isolate classified as non-mucoid by phenotype and thin/non-capsulated by the density-dependent gradient test. No capsule was visible in ATCC 19606, but fibers were observed in the background of the images, most likely representing artifact from sample preparation (Fig. 6 and Table 1). The difference in capsule thickness between the thin/non-capsulated group and the group suspected to be capsulated was significant (*P* < 0.001). Capsule presence was confirmed by India ink staining. The cells appeared purple surrounded by a clear halo on a dark background, indicating a capsule, similar to the CAP-AB isolate. No halo was observed for the thin-capsulated ATCC 19606 (data not shown).

### Capsule visualization in isolates with a discrepancy between phenotype and the density-dependent gradient test

**Fig. 7:**
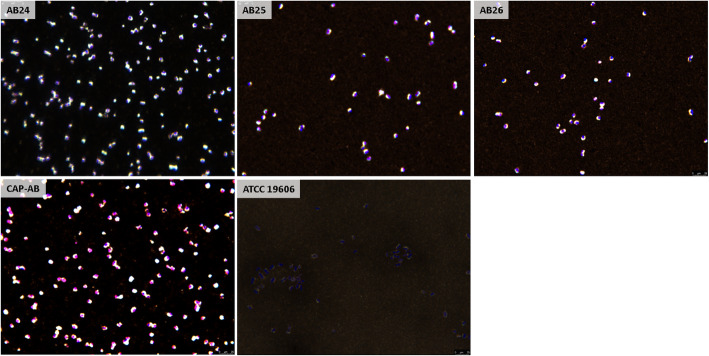
Capsule visualization of the isolates with a non-mucoid phenotype but capsulated according to the density-dependent gradient test. Brightfield microscopy of isolates AB24, AB25, AB26, CAP-AB and ATCC 19606, stained by India ink and crystal violet. Capsule was visible as a clear halo surrounding bacterial cell stained in violet. Images depict the trend observed in three different blood agar plates inoculated with the same isolate

For the three isolates with a non-mucoid phenotype but suspected to be capsulated according to the density-dependent gradient test (AB24, AB25, AB26), capsule presence was confirmed by India ink staining. A significant capsule was observed for CAP-AB, while no capsule was detected for ATCC 19606 (Fig. 7).

## Discussion

To our knowledge, this is the first report of a simple method to confirm capsulated *A. baumannii* based on density-dependent gradient testing. We validated our method by comparing the results to TEM imaging and measurement of carbohydrate production. Our test can verify the presence of a capsule in suspected isolates exhibiting a mucoid phenotype. The test can also detect capsulated strains that do not exhibit a mucoid phenotype. Furthermore, isolates with a heterogeneous production of capsules can be detected using this test. Phase variation, a known phenomenon in *A. baumannii*, modulates capsule exopolysaccharides as well as other phenotypes such as motility, cell shape, biofilm formation, antimicrobial resistance, and virulence [11, 22].

India ink staining is commonly used in clinical microbiology laboratories for capsule visualization. However, this method is labor intensive and requires two hours to prepare a sample. In comparison, our test is quick: a sample can be prepared in a few minutes and results can be read after 30 min.

Identifying capsulated and mucoid *A. baumannii* is clinically and epidemiologically important because the presence of a capsule is associated with virulence. The role of capsules in virulence mechanisms and clinical outcomes is well known for *K. pneumoniae* [10, 23, 24], while data are limited for *A. baumannii* [5, 11]. Only two studies linked *A. baumannii* mucosity and capsule formation to clinical severity and epidemic potential of the strains [12, 25].

Our study has some limitations. First, for simplicity, and in order to reduce the time required to prepare and perform this test, we used a two-phase gradient matrix. Due to this configuration, it is not possible to distinguish between an isolate with a thin capsule and a non-capsulated isolate, since they both migrate to the bottom phase. Higher resolution may have been achieved by adding more phases. Second, results can be influenced by changes in cell density due to different growth conditions, such as media type, temperature, presence of CO_2_ or presence of antibiotics. Third, we did not evaluate the impact of centrifugation time and centrifugal force on the test results.

## Conclusions

Density-dependent gradient testing is a rapid, simple-to-perform, and objective method for confirming capsulated *A. baumannii*. This method can be easily applied for suspected strains with a mucoid phenotype. Identifying capsulated and hypervirulent strains is important for clinical care and for outbreak investigations, for which it may help direct infection control measures.

## Methods

### Sample and phenotype determination

A total of 57 unrelated non- duplicate *A. baumannii* isolates from different hospitalized patients were selected randomly from the collection at the National Center for Infection Control and Antibiotic Resistance in Israel. The sample was composed of isolates collected from 15 medical centers from Israel and Europe in the years 2008–2019. The specimens were isolated from blood (*n* = 25), sputum (*n* = 11), skin (*n* = 8), rectum (*n* = 4), tracheal aspirate/bronchoalveolar lavage (n = 4) and other sites (*n* = 5). The isolates were identified to the species level by VITEK® MS (bioMérieux SA, Marcy l’Etoile, France). Further confirmation of the species was performed by molecular genotyping using methods described by Evans et al. [26]. In brief, typing of *A. baumannii* isolates was conducted by the alignment of OXA-51-like β-lactamase sequence relationships using OXA-69A and OXA-69B primers. Clonality was determined by sequencing of the OXA-51-like gene to assign isolates to international clonal complexes [27]. Of the 57 isolates, ten belonged to clonal complex 2, 33 isolates belonged to clonal complex 3, and 14 belonged to other clonal complexes (Table 1).

All isolates were categorized by phenotype observation as mucoid or non-mucoid. Phenotype was determined following overnight incubation at 35 ± 2 °C on blood agar (tryptic soy agar supplemented with 5% sheep blood; Hylabs, Rehovot, Israel). Three clinical microbiologists evaluated the phenotype according to several colony morphology parameters, including texture, elevation, margin, size and shape [28]. If the categorization by the three microbiologists was not identical, the isolate was excluded (Table 1).

We also included in the sample three well-characterized specimens as controls: (i) a non-mucoid and thin-capsulated *A. baumannii* (ATCC 19606); (ii) a mucoid *A. baumannii* with a significant capsule (CAP-AB). This isolate harbors a capsule locus type KL17 including wza-c genes and three different glycosyltransferases gtr38, 39, 40. (iii) A hypermucoid string test-positive *K. pneumoniae* (HM-KP).

All isolates were stored at − 80 °C before sub-culturing and analysis.

### Principle of the density-dependent gradient method

The method is based on bacterial cells passing through a gradient matrix as a result of centrifugal force. The gradient was composed of multiple phases, each phase with a different concentration (20–80% V/V) of colloidal silica particles of 15–30 nm (Percoll, GE Healthcare, Uppsala, Sweden). Bacterial cells were inoculated to the top of a gradient and, due to centrifugation, migrated to different locations within the density gradient based on capsule size. This method assumes that cell size is similar between *A. baumannii* strains.

### Validation of gradient composition

The validation was first performed on the three control strains and then confirmed on six isolates selected randomly from the sample: four with a mucoid phenotype and two with a non-mucoid phenotype. The nine isolates were prepared by inoculating a single colony in 10 mL BHI (Hylabs, Rehovot, Israel). The cultures were incubated for 18 h at 35 ± 2 °C with shaking (250 rpm), to reach a stationary growth phase (final normalized optical density OD_600_ of 0.7–0.9). The cultures were centrifuged for 10 min at 3200×*g* and the bacterial cell pellet was re-suspended with sterile phosphate buffer saline (PBS; Bio-Lab, Jerusalem, Israel) to a final volume of 2 mL.

In order to achieve robust separation and easy visualization of bacteria in the gradient matrix, we determined the optimal silica concentrations of the phases and the minimum number of phases required. To determine the optimal silica concentrations, a panel of seven single concentrations of silica (20, 30, 40, 50, 60, 70, 80% V/V) with a final volume of 500 μL were prepared. An aliquot of 100 μL of each of the nine isolates was placed on the top of each tube and was centrifuged for 10 min at 8000×*g*. The migration of the bacterial band was visually examined in the seven tubes and concentrations with bands that yielded a clear band within the gradient, without any smears, were selected. We rejected tubes in which bacterial cells could not penetrate the matrix or bacterial bands had settled at the bottom of the tube. The minimum and maximum silica concentrations of the remaining tubes composed the range of work.

### Gradient matrix preparation

The matrix was prepared as follows: 1 mL of the lower density silica concentration was placed in an empty round-bottom tube (12/75 mm, Heinemann Labortechnik GmbH, Duderstadt, Germany). Using a disposable syringe (23G needle), 1 mL of the higher density concentration was inserted at the bottom of the tube, allowing the lower density concentration to rise above it.

### Sample preparation and analysis

We used two techniques to prepare the samples: (i) bacterial cells from BHI broth as described above in the “validation of gradient composition” section. Following centrifugation, the cell pellet was re-suspended for a final volume of 1 mL. (ii) Bacterial cells directly from plate: a 5 μL loop-load of colonies from an overnight incubation on blood agar (35 ± 2 °C) was suspended in 1 mL PBS. In order to evaluate whether the growth media affected the results, all tests were performed in duplicates from BHI broth and from blood agar plates.

An aliquot (600 μL) of the bacterial cells was inoculated to the top of the gradient matrix. Following centrifugation for 30 min at 3000×*g*, results were read by measuring the height of the bacterial band from the liquid level down to the middle of the bacterial band. A bacterial band that migrated to the bottom phase of the gradient was classified as a thin or non-capsulated strain, while a bacterial band that concentrated in the top phase was suspected to be a capsulated strain.

### Total carbohydrate quantification

This test is based on the reaction of carbohydrates with sulfuric acid, which produces furfural derivatives that develop a detectible color when reacting with phenol [29, 30]. The test quantifies the total polysaccharides present in the bacterial cell, including capsular polysaccharides and LOS. Extraction was performed by centrifuging 1 mL of a culture for 5 min at 14,500×*g* and washing the cells with 1 mL of 50 mM NaCl five times. During the last washing cycle, the cells were re-suspended in 50 mM ethylenediaminetetraacetic acid to normalize the solution to OD_600_ 2.0. The sample was incubated at 35 ± 2 °C for 1 h in order to release the polysaccharides from the cell. Following centrifugation, 200 μL of the supernatant of each isolate was used for quantification. A range of known sucrose/fructose (1:1 W/W) concentrations (0–240 μg/mL) was used to construct a standard curve. 200 μL of 5% phenol (in water) and 1 mL of 93% sulfuric acid were added to the samples and to the standards. Furfural derivatives were measured by optical absorbance at 490 nm (OD_490_) after 10 min. The total carbohydrate amount was calculated from the standard curve.

### Tem

Six isolates selected randomly from the sample were imaged: one isolate determined to be thin or non-capsulated by the density-dependent gradient test, three isolates suspected to be capsulated by the density-dependent gradient test, and the two *A. baumannii* controls. The samples were prepared according to a standard protocol [31]. An aliquot of 1 mL of a culture grown overnight in BHI medium at 35 ± 2 °C with shaking was centrifuged and the supernatant was removed manually. The bacterial cells pellet was re-suspended and fixed for 3 h in Karnovsky mixture [31]. To remove fixative residues, the cells were washed three times with 0.1 M sodium cacodylate buffer. The cells were post-fixed in 1% OsO_4_, 0.5% K_2_Cr_2_O_7_, 0.5% K_4_[Fe (CN)_6_] and stained with 2% uranyl-acetate. The samples were washed with distilled water, dehydrated using ethanol and embedded with resin (Epon EMBED 812, EMS, Hatfield, PA) and polymerized at 60 °C for 24 h. Ultrathin sections (90–70 nm) were obtained using an ultra-microtome (EM UC7, Leica Biosystems, Buffalo Grove, IL). Samples were imaged using a TEM at 120kv (G-12 Spirit, FEI, Hillsboro, OR). Capsule thickness was determined by measuring five different sites on each isolate. For each isolate, the mean and standard deviation of the five measurements were calculated.

### India ink staining

The samples were prepared according a standard protocol [15]. Briefly, a drop of India ink (Black 17, Pelikan, Hannover, Germany) was placed on a microscope slide and mixed with a colony from an overnight culture on blood agar. The slide was then dried under air at room temperature for 30 min. The slide was saturated with 1% crystal violet (Merck, Rehovot, Israel) for 1 min, rinsed with deionized water and dried under air at room temperature for 2 h. Observation of the slide was performed with a light microscope (Leica LMD7, Leica Microsystems, Buffalo Grove, IL).

### Statistical analysis

We used a Student’s t-test to compare total carbohydrates and capsule thickness between the capsulated and thin/non-capsulated isolates.

## Data Availability

The datasets used and/or analyzed during the current study available from the corresponding author on reasonable request.

## Abbreviations

LOS: lipooligosaccharides
PNAG: poly-β-(1–6)-N-acetylglucosamine
HM-KP: Hypermucoid string test-positive *K. pneumoniae*
CAP-AB: Capsulated and mucoid *A. baumannii*
BHI: Brain heart infusion broth
TEM: Transmission electron microscopy
PBS: Phosphate buffer saline
